# Some Social Factors That Tend to the Degeneration of the Race and Alcoholism

**Published:** 1903-09

**Authors:** R. R. Kime

**Affiliations:** Atlanta, Ga., Ex-President Georgia State Sociological Society; Ex-President Tri-State Medical Society of Alabama, Georgia and Tennessee; Ex-President Atlanta Society of Medicine; Member American Medical, Southern Surgical and Gynecological, Tri-State Medical, Georgia State Medical Societies, etc., etc.


ATLANTA

### Journal-Record of Medicine.

Successor to Atlanta Medical and Surgical Journal, Established 1855, and Southern Medical Record, Established 1870.

VOL. V. SEPTEMBER, 1903. No. 6.

BERNARD WOLFF, M.D., EDITOR, Nos. 319-20 Prudential.

M. B. HUTCHINS, M.D., BUSINESS MANAGER, 1007-1008 Century Bldg.

Published Monthly.

### ORIGINAL COMMUNICATIONS.

### SOME SOCIAL FACTORS THAT TEND TO THE DEGENERATION OF THE RACE AND ALCOHOLISM.*

BY R. R. KIME, M.D., ATLANTA, GA.,

Ex-President Georgia State Sociological Society; Ex-President Tri-State Medical Society of Alabama, Georgia and Tennessee; Ex-President Atlanta Society of Medicine; Member American Medical, Southern Surgical and Gynecological, Tri-State Medical, Georgia State Medical Societies, etc., etc.

In considering the future of our race we have but two conditions to study, improvement or degeneration, since there is no such thing as an absolute standstill without degeneration.

The attributes and faculties of a people, without use, tend to decay and degenerate. If used they tend to uplift or degrade, according as their work is good or evil, right or wrong.

You may inquire what is right or wrong action, and by what principle shall we be guided in making our decision? I answer:

*Paper read by title at Georgia State Sociological Society, Savannah, June, 1903

"That which tends to perpetuate, elevate and improve the race is good, and that which tends to degrade, degenerate and destroy the race is evil."

Laying aside the question of religion, then, as an individual, community, State or nation, we must decide whether our actions tend to uplift or degrade, and act accordingly. We are responsible for our actions not only as individuals, but as members of the home, residents in a community and citizens of the State and nation.

Our right or wrong actions not only affect us as individuals, but affect the home, community, State and nation, and are often visited to the second and third generations after us. Hence it is very important that our every action be in line with the development and improvement of the race.

Individual action and individual liberty imply individual responsibility, not alone to ourselves, but to those who come after us.

If we foster, encourage or engage in evil we injure ourselves, degrade those with whom we are associated, and place our offspring in bondage. Thus, ofttimes by heredity, environment and training the child is doomed to criminality, insanity or degeneracy.

All men are not born equal, and can not be so long as the parents are imperfect. Absolute freedom and equality require absolute purity of character—physical, mental, moral and spiritual. The fruit is but an index of the worth and character of the tree; so with the individual.

If we desire our children to be born free and equal, we must not engage in anything that is evil, or that tends toward evil; neither must we encourage that which is evil in others if we would do our duty to ourselves and humanity.

This is not a question of religion or sentimentalism alone, but one of justice, as well as one of great importance to us as a people, from a business and social standpoint.

It is far easier and less expensive, to say nothing of the attendant suffering and sorrow, to prevent the development of the criminal, the insane, the degenerate, the inebriate, etc., than to care for and protect them.

We claim to be an enlightened, intelligent nation, yet we foster and encourage, license and protect evils that we know tend to degenerate and destroy the race. We take money procured at the sacrifice of human souls, blood money that carries with it a curse, and use it, expecting such to redound to our name's honor and glory. Will funds thus secured tend to elevate and perpetuate the race? If not, it is suicide for us to so continue.

If a thing is wrong and destroys that which is noble and best in mankind, by what principle of morality can such a thing be licensed and made right?

If we desire to build up good physical, mental, moral and spiritual characters, individually and as a people, we must eliminate that which is evil, not alone from a religious and moral standpoint, but from a physical and intellectual standpoint as well.

We, as Americans, are living at too rapid a rate. In the mad rush for financial, intellectual and so-called social supremacy, the physical, moral and spiritual are sacrificed.

In the hustle to keep pace with the procession we have no time for rest, recreation and building up of the physical, and this continues until a breakdown comes and we are compelled to do so.

The child is placed in school at the age of six, pushed to its utmost capacity to keep up with its classes, and kept in school from 8:30 a.m. until 1:30 to 2 p.m. in cities. Many are hurried to school of mornings with but little, if any, breakfast, and do not get their midday meal until 2 to 3 p.m. This means four to five hours hard study, breakfast at 8 a.m., dinner meal at 2:30 p.m.—six and a half hours between meals. Can children do good work, and improve physically and mentally as they should under such a system?

In many instances children are fed on improper food, and as they grow older, especially girls, dressed improperly, are up late of nights, taxed with learning extra accomplishments and with the customs of society. With all this no wonder many are physical wrecks before fully matured, and unfit for motherhood and the duties of life.

Children thus reared in the vitiated air of cities often deteriorate as much physically as others of the more unfortunate class from child labor.

To this audience it is only necessary to mention the subject of child labor as a factor in the degeneration of the race.

One thing we must not forget in relieving these helpless children of the burden of child labor: we must prepare for their education and see that they receive it.

Any bill that regulates or prevents child labor without preparation for and requiring the education of such children falls short of what it should do, and indirectly may add to indolence and crime.

Sociologists, neurologists, alienists and economists tell us that, in many instances, crime, insanity, mental degeneracy, alcoholism, pauperism, etc., are due in a great measure to heredity; and yet we, as a people, in the presence of the light of knowledge, fail to utilize that knowledge for the benefit of humanity. Ministers of the gospel and officers of the law, by legal authority of the State, unite in marriage such persons as they know are morally and physically unfit for such relations in life.

I ask, in the name of humanity and justice, is it right to unite in marriage such parties as we can not help but know will bring helpless victims into the world to suffer at times worse even that death itself? If they are physically diseased and incurable, their offspring will be degenerated and diseased. Such persons, for the sake of humanity and the upbuilding of the race, should refrain from or be restricted in marriage.

Those persons who have by wilful evil acts of their own destroyed that which is noblest and best in their natures, and have become habitual criminals or paupers, should not be allowed to marry and propagate their kind by sanction of the law. It is argued this will increase illegitimacy. I ask does the habitual criminal recognize and obey the moral obligations of the marital vow? Can we, as an enlightened people, afford to sanction and legalize evil that brings helpless victims into a bondage worse than slavery itself, to be a care and menace to the State, simply to prevent what might occur, and that an evil of itself?

To such I would say there is a remedy which is just and humane and in keeping with the dictates of humanity. Render them sterile. If we as a people would use a little more common sense and judgment, and not be guided solely by sentimentalism on the subject of marriage, there would be less crime, vice, disease and divorce scandals.

Marriage, the most important relation in life, fraught with so much peace and happiness, or with so much evil and sorrow, is looked upon by many as a trivial affair, and entered into with less rational consideration, thought and judgment than many of the ordinary business affairs in life.

Marriage and marriage vows should be held more sacred and not degraded to a matter of convenience, the propagation of evil, to be directed and controlled by fickle sentimentalism, without judgment and discretion.

Such marriages as these tend to degenerate and exterminate the race by producing deseased and degenerate offsprings that become a menace, burden and expense to the community and State. They are the kind that fill our almshouses, jails, reformatories, etc.

The best remedy in such cases is prevention. I do not discourage matrimony by any means, but urge giving all possible encouragement to the fit and properly developed, but as to the unfit and diseased, humanity and the development of the race speak for itself.

I do not recommend any extreme radical means that might interfere with social purity, but there are reasonable, rational re strictions that can be utilized with great benefit to mankind.

Physicians, teachers, lawyers, and especially ministers, can do much in educating and moulding public opinion along these lines. Can we do our duty to humanity and neglect such opportunities for doing good?

Another growing evil of the day and generation, especially in cities, is the avoidance of maternity by sacrifice of life, a crime looked upon by many as a light, trivial matter.

The public conscience needs to be aroused and lifted to higher conceptions of what is right and wrong on this question.

The customs of society and the demands of the fashionable are responsible for much of this evil. The purity of the home, the foundation on which the State must rest, is being jeopardized by the demands of society that interfere with the home-life and home-training of children.

Fashionable gambling, fashionable drinking and kindred vices can not help but have their influence on the home and youths of our land.

It is with fear and trembling that I view an increased tendency on the part of women to engage in such evils. Heretofore the purity of the home and the salvation of the race have been maintained by the purity of women.

We should all unite, men and women, regardless of creed or profession, in condemning that which assails the purity, sanctity and perpetuation of the home, and that which tends to degrade humanity.

We all recognize the evils of the saloon in producing degeneration of the race and alcoholism. It was hoped that with the temperance organizations, church influence, prohibition and local option forces at work there would be a decrease in the consumption of liquor.

We are informed that the U. S. Treasury statistics show an alarming increase which has been growing since 1896.

"During the last fiscal year the drinking classes of this country consumed forty (40) gallons of wine, beer and spirits per capita, or, say, twenty gallons for every man, woman and child in the Union."

"It also shows that the increased consumption of light liquors is not accompanied by a corresponding decrease in the use of spirits. Indeed, the direct opposite is proven to be true."—*The New Voice*, March, 1903.

"In 1900, 1,349,176,033 gallons consumed; 17.68 gallons per capita.

In 1901, 1,390,127,379 gallons consumed; 17.90 per capita.

In 1902, 1,539,081,991 gallons consumed; 19.48 per capita."

Something is radically wrong. What can we hope for as a Nation so long as this ratio of increase exists? Sociologists tell us crime is on the increase. (Ellis, The Criminal, p. 259.) "In France, in Germany, in Italy, in Belgium, in Spain, in the United States, the tide of criminality is becoming higher steadily and rapidly."

Dr. Brower says: "We are surprised at the rapid increase of crime, rapid much beyond the increase of population." In addition to this, the medical profession recognizes an increase in insanity and tuberculosis.

Shall we shut our eyes and lull ourselves into a false hope of security while Satan's hosts march bravely on? Listen, McDonal says: "The saloon is the true home of the criminal. Of 100,000 murders committed in France, 2,347 occurred in saloons. Out of 49,423 arrests in New York, 30,507 were drunkards."

Brower: "Fully 50 per cent. of the criminals arrested in Chicago are inebriates. Alcohol is the direct or indirect cause of probably 75 per cent. of all crime committed."

Ellis: "Alcoholism in either of the parents is one of the most fruitful causes of crime in the child."

Dr. Stern: "At least 70 per cent. of all perpetrated crime is due directly or indirectly to alcohol."

Dr. Brower: "Alcohol is the direct or indirect cause of probably 75 per cent. of the crime committed."

Dr. N. S. Davis, father of the American Medical Association, states: "That the daily or habitual use of alcoholic drinks, even in moderate doses, not only impairs both health and morals and leads to slow tissue degenerations, but also impairs the metabolic and nutritive processes, so as to render the individual more liable to attacks of all acute infectious diseases, and more liable to die when attacked, is a fact which has been fully demonstrated not only in the laboratory of the scientists, but also in every field of human labor and in every variety of climate."

Dr. Powell writes: "From the best evidence obtainable the most potent factor in the causation of defective mental action is alcoholism."

"While there is a very small per cent. of insanity and other deplorable maladies due directly to alcoholism, a very large per cent. is indirectly due to it. Many alienists who have given much attention to the subject, put it from 50 to 75 per cent.

"It is evident that to lessen drunkenness is to lessen insanity and other morbid conditions. There can not be any doubt but that children of intemperate parents are much more liable to insanity, epilepsy, idiocy, imbecility, intemperance, etc., than are those of temperate parents."

"The worst effects of alcoholism are to be found visited upon the succeeding generation of drunken parents. If the habits of women were like those of the men we would have degenerated long ago."

Same author: Of all the nervous diseases which we inherit there are none more disastrous than alcoholism."

Blanford, one of the standard authors on insanity and its treatment, says: "If we could ascertain the statistics of insanity in other countries, civilized and semi-civilized, I think we should find insanity in proportion to the use of intoxicating liquors and substances."

Dr. Peter Brice, a distinguished alienist, states: "Nine tenths of insanity is due to hereditary taint. That 75 per cent. of this hereditary taint is due to intemperance in the ancestors is not exaggerated."

He further states: "I know of nothing so potent in the production of brain degeneration as alcohol."

"In the prophylaxis against criminality we should try to check alcoholism. In all countries the growth of viciousness and of aberration of the moral sense goes hand in hand with the growth of alcoholism and of other poisons. Why are so many crimes committed under alcoholic influence? Why are so many criminal recidivists of inferior intellect and morality? Experimental physiology and psychology give us the answer. Alcohol produces a progressive degenerative effect on the cortical nerve-cells, as well as on the other nerve elements which take part in the intellectual and moral functions. Continuous indulgence in alcoholic drinks reduces the general as well as the special vitality of the tissues; the faculties are impaired, and abnormal character and acts result. This takes place in normal beings. Should the subjects be hereditarily predisposed, the result is still worse."—*Dr. Jul. Morel, Chief Physician State Insane Asylum, Mons, Belgium.*

"The municipal authorities of Glasgow, according to the *Lancet*, recently appointed a special committee to inquire into and report on the effect of alcoholic drinks on the increase of lunacy, which has become a serious matter in that municipality and in Scotland generally. A copy of the report, our contemporary says, furnishes serious matter for consideration. Out of 565 admissions to the Glasgow District Asylum and 213 admissions to observation wards of the poorhouses during twelve months, no less than 33 per cent. were traceable to alcoholic drinks as a cause. The inquiries show that want and privation have not led to insanity, nor were they causes of alcoholic drinking, for the cases thus admitted were from all classes and conditions of society, and in the majority the home surroundings, conditions and earnings were good. These are not isolated facts, for the figures of the Royal Edinburgh Asylum point in the same direction."—*Amer. Med. Assoc. Journal, June 20, 1903.*

But this is sufficient to demonstrate what alienists, neurologists and sociologists believe to be true in regard to alcohol, and this applies only to disease and degeneration resulting from its use. To these add the millions spent for drink, the amount of pauperism, the heartaches and suffering of helpless women and innocent children, the wrecked homes, and last but not least, the moral degeneration of the race, and what a picture for a civilized nation to contemplate! Can the human mind comprehend this picture and fathom of its meaning?

Something is radically wrong, some one is responsible. Is it you and I? I answer, yes, we are either by sins of omission or commission, guilty. We as a State and nation furnish the seed and the soil; hence we must reap what is sown in it.

It is also self-evident to the reasoning mind that a State and nation is what its citizens make it. Each has character as well as individuals, and each individual helps to form that character; and character is not developed by chance but by fixed laws.

Good from good grows, evil from evil flows. If evil exists there is a cause, and it is your duty as well as mine, to search for and remove that cause, regardless of creed, profession or station in life. Anything short of this is duty left undone.

In no age of the world have we needed manly men and womanly women more than at present; people with character, "grit" and backbone that will recognize and condemn vice and evil in high as well as in low estate; people that are willing to make some personal sacrifice of individual, personal comfort and pleasure for the good of humanity; people that recognize the common brotherhood of humanity, and that are willing to make an earnest effort to ferret out and remove as far as possible the cause of crime, vice and disease.

This leads us to inquire what are some of the causes of alcoholism? The customs of society, the relation of the churches and medical profession, are responsible for 95 per cent. of the acquired intemperance of to-day.

The erroneous views as to the medical, physiological and food effects of alcohol that have been promulgated from time immemorial have done much harm and wrecked many lives.

It is only within the last few years that physicians, scientists and investigators are coming to a better and proper understanding of the effects of alcohol on the human body. Much that was taught formerly was error, clothed with the dictates of authority.

Many leading physicians of the day are recognizing that alcohol is no longer an indispensable necessity in the treatment of disease. Many cases of typhoid fever, pneumonia, diphtheria, scarlet fever, septicemia, shock, surgical operations and abdominal sections are treated as efficiently, if not more so, without than with alcoholics.

Understand, I am not yet prepared to say alcohol has no place in medicine, but I firmly believe the day is coming when it will play a very minor part in the treatment of disease, and the physician of to-day, who will not take the pains to inform and equip himself with other remedies and means at his command, that are equally as efficient and less deleterious in most diseases, is not doing his duty to humanity. The physicians that indiscriminately and thoughtlessly prescribe alcoholics, opiates, cocain, etc., are doing much to injure the race, and should be avoided by those who desire to better social conditions.

What about you, my sister or my brother? What are you doing with the whisky bottle in your home? Is it a panacea for all ills and resorted to for every ache and pain? Do you dose your children with it for every complaint, and do you drench your tiny tender baby with whisky toddies for every cry, without searching for the cause of its discomfort? If you do, you are responsible by precept and example for much of the intemperance of to-day.

Do you dose yourselves and children with all kinds of patent medicines, nostrums, and your baby with soothing syrup, the composition of which you know nothing? If you do, listen:

Dr. Bumgardner, in Transactions Colorado State Medical Society, 1902, reports the percentage of alcoholics in the medicines named as follows:

| | |
|---|---|
| "Greene's Nervoura, 17.2. | Hood's Sarsaparilla, 18.8. |
| Brown's Iron Bitters, 19.7. | Paine's Celery Compound, 21.0. |
| Ayer's Sarsaparilla, 26.2. | Warner's Safe Tonic Bitters, 35.7. |
| Parker's Tonic, 41.6. | Hostetter's Stomach Bitters, 44.3. |

He farther states: "A two-ounce bottle of Mrs. Winslow's Soothing Syrup contains one half grain of morphine; one ounce of Dr. Bull's Cough Syrup contains more than one fourth grain. Also that certain consumption cures are now put in small bottles because not being a permanent mixture, the last dose from large bottles sometimes kills the patient, but that the last dose from smaller bottles would contain too little morphine and cannabis indica to prove fatal." (American Medicine.)

"We are also informed that most morphine and whisky cures contain morphine and whisky, or both."

"Massachusetts Board of Health found alcohol in Peruna, 23.46 per cent. by weight; 16.77 per cent. in Lydia Pinkham's Vegatable Compound; 15.33 per cent. in Vinol, and 5.87 per cent. in Swamp Root."

"H. P. Hynson (American Phar. Association) reports importations since 1898: Cocaine increased 400 per cent.; morphine and opium increased 600 per cent., while the medical uses are probably lessened."

And yet with all this, many good people continue to foster the whisky bottle, and use patent nostrums, while the "good pastor," for the sake of humanity, gets his name in the paper recommending some cure-all or some "quack doctor," as God's messenger to his flock, and these recommendations are sometimes even found in Church papers. We can understand why secular papers will print such advertisements and also of remedies for "Men" and "Women"

—I hope you can read between the lines—that are a disgrace to a civilized much less a Christian nation. It is dollars and cents—but church papers should have a higher moral standard.

It is no uncommon thing of late to see a half-page "ad" in the daily papers with pictures of prominent officials, lawyers and women, some even prominent temperance workers, recommending "Peruna," which is reported to contain 23.46 per cent. of alcohol by weight.

Temperance reformers can well afford to turn their attention to such patent nostrums that contain so much alcohol.

Another thing that deserves their consideration are the beverages at soda founts. One popular drink contains cocain, caffein, and a small percentage of alcohol.

It is estimated 28751 grains of cocain were consumed in Atlanta in one year, 1901, in this harmless beverage, and 75 per cent. of that by boys and girls, young men and young women. Since 1901 its use has probably increased. Another, if its name indicates its character and effect, is a disgrace and shame to any decent community.

Comment is unnecessary. I leave you to meditate and consider your own individual influence in these matters.

